# Multiple facets of stream macroinvertebrate alpha diversity are driven by different ecological factors across an extensive altitudinal gradient

**DOI:** 10.1002/ece3.4841

**Published:** 2019-01-15

**Authors:** Zhengfei Li, Xiaoming Jiang, Jun Wang, Xingliang Meng, Jani Heino, Zhicai Xie

**Affiliations:** ^1^ The Key Laboratory of Aquatic Biodiversity and Conservation, Institute of Hydrobiology Chinese Academy of Sciences Wuhan China; ^2^ University of Chinese Academy of Sciences Beijing China; ^3^ State Key Laboratory of Eco‐hydraulic in Northwest Arid Region of China Xi’an University of Technology Xi’an China; ^4^ Biodiversity Centre Finnish Environment Institute Oulu Finland

**Keywords:** ecological drivers, functional diversity, highland streams, macroinvertebrates, phylogenetic diversity, taxonomic diversity

## Abstract

Environmental filtering and spatial structuring are important ecological processes for the generation and maintenance of biodiversity. However, the relative importance of these ecological drivers for multiple facets of diversity is still poorly understood in highland streams. Here, we examined the responses of three facets of stream macroinvertebrate alpha diversity to local environmental, landscape‐climate and spatial factors in a near‐pristine highland riverine ecosystem. Taxonomic (species richness, Shannon diversity, and evenness), functional (functional richness, evenness, divergence, and Rao's Quadratic entropy), and a proxy of phylogenetic alpha diversity (taxonomic distinctness and variation in taxonomic distinctness) were calculated for macroinvertebrate assemblages in 55 stream sites. Then Pearson correlation coefficient was used to explore congruence of indices within and across the three diversity facets. Finally, multiple linear regression models and variation partitioning were employed to identify the relative importance of different ecological drivers of biodiversity. We found most correlations between the diversity indices within the same facet, and between functional richness and species richness were relatively strong. The two phylogenetic diversity indices were quite independent from taxonomic diversity but correlated with functional diversity indices to some extent. Taxonomic and functional diversity were more strongly determined by environmental variables, while phylogenetic diversity was better explained by spatial factors. In terms of environmental variables, habitat‐scale variables describing habitat complexity and water physical features played the primary role in determining the diversity patterns of all three facets, whereas landscape factors appeared less influential. Our findings indicated that both environmental and spatial factors are important ecological drivers for biodiversity patterns of macroinvertebrates in Tibetan streams, although their relative importance was contingent on different facets of diversity. Such findings verified the complementary roles of taxonomic, functional and phylogenetic diversity, and highlighted the importance of comprehensively considering multiple ecological drivers for different facets of diversity in biodiversity assessment.

## 1 INTRODUCTION

Disentangling the ecological drivers shaping biodiversity patterns is of primary importance in ecology, biogeography, and conservation biology (Loreau et al., 2001; Tilman et al., 2001). Traditionally, emphasis has been on the relationships between diversity patterns and environmental variables, and numerous studies have illustrated that biodiversity is hierarchically regulated by different environmental factors ranging from local through landscape to regional scales (Corbelli et al., 2015; Macedo et al., 2014; Ricklefs, 1987; Strecker et al., 2011). In recent years, it has also been increasingly recognized that besides environmental variables, spatial factors related to species dispersal and other stochastic forces play important roles in shaping biodiversity patterns, which may decrease the match between community composition and environmental conditions (Heino, Melo, et al., 2015).

The majority of relevant studies have typically focused on elucidating the effects of both multiscaled environmental and spatial factors on taxonomic composition and diversity (Beisner, Peres, Lindstrom, Barnett, & Longhi, 2006; Munemitsu & Noriko, 2011). However, taxonomic diversity treats species as functionally equivalent and evolutionarily independent (Saito, Siqueira, & Fonseca‐Gessner, 2015), and thus neglects the differences in functional features and evolutionary relationships between species which may help in providing a more comprehensive picture of community assembly (Botta‐Dukát, 2017; Schmera, Heino, Podani, Erős, & Dolédec, 2017). Consequently, ecologists have extended the examination of community assembly from purely taxonomic to functional and phylogenetic diversity measures (Mason, Mouillot, Lee, & Wilson, 2005; Webb, Ackerly, McPeek, & Donoghue, 2002). Overall, functional diversity captures the value and range of organism traits that influence their performance and ecosystem functioning (Mason et al., 2005; Villéger, Mason, & Mouillot, 2008). In contrast, phylogenetic diversity refers to the evolutionary relationships among species, and it should thus reflect the imprints of evolutionary and biogeographic history on community structure (Feng et al., 2014; Webb et al., 2002).

According to evidence from some recent studies, taxonomic, functional, and phylogenetic diversity should respond to distinct ecological drivers, implying that different facets of diversity may be complementary and provide integrative information about community assembly (González‐Maya, Víquez‐R, Arias‐Alzate, Belant, & Ceballos, 2016; Heino & Tolonen, 2017b; Saito, Siqueira, et al., 2015). Generally, taxonomic diversity might be affected by both deterministic (e.g., environmental conditions) and some stochastic processes (e.g., dispersal and ecological drift) (Munemitsu & Noriko, 2011). This is understandable as the absence of a species in a particular habitat can either be owing to it was not dispersed there or because the environmental conditions were unsuitable for it to survive despite it had reached the habitat (Heino et al., 2017). Besides, some processes related to historical factors may also strongly structure regional species pool and thus influence species diversity within localities (Feng et al., 2014). Functional diversity should be most strongly determined by environmental variables owing to the close association between functional traits and environmental conditions (Ding et al., 2017). The responses of phylogenetic diversity may be more complicated and scale dependent. At small spatial scales, variation in phylogenetic diversity is probably determined by environmental conditions along with functional diversity owing to evolutionary conservatism (Heino & Tolonen, 2017a). In contrast, at larger spatial scales, dispersal limitation and historical processes may play more important roles for variation in phylogenetic diversity (Morlon et al., 2011).

To date, the investigations involving ecological drivers for multiple facets of alpha diversity of freshwater communities have mainly been carried out in lowland streams, lakes, or ponds (Heino & Tolonen, 2017b; Saito, Siqueira, et al., 2015; Tolonen, Vilmi, Karjalainen, Hellsten, & Heino, 2017). Such studies in highland or alpine streams are still largely lacking and mostly centered on examining taxonomic diversity (e.g., Jacobsen, 2008; Jiang, Xie, & Chen, 2013; Kock Laursen, Hamerlik, Moltesen, Seestern Christoffersen, & Jacobsen, 2015). Alpine regions and streams have attracted increasing interest in recent years owing to the more pristine conditions than in lowland areas, and the high vulnerability to various environmental threats, such as a series of challenges posed by global warming (Tong & Wu, 1996). These stressors could have prominent effects on stream biota, especially for those with high sensitivity to environmental changes including benthic macroinvertebrates (Harper & Peckarsky, 2006; Inouye, Barr, Armitage, & Inouye, 2000). Consequently, understanding the drivers of diversity patterns of stream macroinvertebrates can improve our knowledge of community and ecosystem response to local and global changes. Increasing such understanding is also a prerequisite for effective conservation planning in the near future. These investigations should therefore facilitate our understanding of the mechanisms underlying the generation, maintenance, and conservation of freshwater biodiversity (Dudgeon et al., 2006).

In this study, we examined the responses of multiple facets of alpha diversity of stream macroinvertebrates to different ecological drivers in Southern Tibet, one of the world's important alpine biodiversity hotspots (Jiang et al., 2013; Tonkin et al., 2017). These sites are presumed to have minimal human alteration, a feature that allowed us to focus on natural gradients of environmental variation. Our main goal was to understand the relationships between the different diversity indices involved, and whether functional and phylogenetic diversity responded to environmental gradients and spatial vectors in a similar way to species richness. In addition, we tested the following hypotheses: (a) the number and identity of key environmental (i.e., landscape‐climate and habitat variables) and spatial factors varied and contributed differently to the variation in each facet of diversity (i.e., taxonomic, functional and phylogenetic diversity); (b) the relative importance of environmental and spatial predictors varied among different facets of diversity. Based on the second hypothesis, we also assumed that environmental variables rather than spatial factors play a dominant role in determining functional diversity, while spatial effects may be more important for the other two facets compared to functional diversity. We hope this study could help understanding how biodiversity patterns are generated and predicting shifts in biodiversity distribution with environmental changes, which may provide guidance and reference for biodiversity conservation in pristine alpine freshwater ecosystems.

## 2 METHODS

### 2.1 Study area

This study was conducted in the Yarlung Zangbo Grand Canyon area (26°52′N–30°40′N, 92°09′E–98°47′E), Southern Tibet (Figure 2). This region is known to have the deepest canyon in the world, and covers a total area of 117,000 km^2^, with complex landforms and a maximum elevation gradient of about 7,000 m. It is rich in freshwaters, being densely covered with rivers, lakes and other forms of water resources such as glaciers. Climate conditions are complex and diverse, ranging from tropical to temperate and arctic zones, possessing a unique ecosystem, with species of animals and plants barely explored and influenced by human activities (Li, Yang, Wang, Zhu, & Tang, 2010).

### 2.2 Macroinvertebrate sampling

Benthic macroinvertebrate samples were taken from 55 stream sites from the tributaries (sub‐basins) of the Yarlung Zangbo River Basin across large geographic and climatic gradients (from subtropical to temperate zones) in October 2015 (Figure 2). These sites were all pristine or near‐pristine, not impacted by notable anthropogenic interference, a feature that allowed us to focus on natural environmental variation. At each site, three quantitative samples were taken along a 100 m reach of a stream in the principal habitats (usually riffles) with a Surber sampler (30 × 30 cm in area, with 500 μm mesh size) and then sieved with a 500 μm sieve in the field. Specimens were handpicked within 5 hr of collection from the sediment on a white porcelain plate and later stored and preserved in 70% ethanol. Macroinvertebrates were identified to the lowest taxonomic level (usually genus or species) where possible in the laboratory, according to the relevant references (Brinkhurst, 1986; Dudgeon, 1999; Epler & Quality, N.C.D.o.W., 2001; Morse, Yang, & Tian, 1994; Zhou, Gui, & Zhou, 2003).

### 2.3 Environmental variables

We measured habitat environmental variables at each site after macroinvertebrate sampling. Channel width (measured using a Ranger Finder instrument) and water depth were averaged from at least five equal cross‐stream transects. Current velocity (ms^−1^) was determined in the middle of the sampling location with a LJD‐10 flow‐meter. Water temperature, pH, and conductivity were measured with an YSI6680 Multiprobe. The percentage of different substratum particle sizes was estimated according to Kondolf (1997). Substratum was assigned into one of the five types: (a) Sand (<2 mm), (b) Gravel (2–32 mm), (c) Pebble (32–64 mm), (d) Cobble (64—256 mm), and (e) Boulder (>256 mm), and their percentages were estimated at each site using a 1 m^2^ grid. We also assessed coarse woody debris (CWD) within each study site as a measure of aquatic habitat complexity (Arnaiz, Wilson, Watts, & Stevens, 2011). CWD was measured using a rapid scoring, where no woody debris scored 0, scattered small quantities scored 1, regular large quantities scored 2, and log jams approximately the width of the stream scored the maximum of 3.

We used altitude and bioclimatic factors (i.e., temperature and precipitation) to represent landscape‐climate variables (Colzani, Siqueira, Suriano, & Roque, 2013). Temperature variables included annual mean temperature, isothermality, temperature seasonality, maximum temperature of warmest month and minimum temperature of the coldest month. Precipitation variables contained annual precipitation, precipitation of wettest month, precipitation of driest month, precipitation of warmest quarter, precipitation of coldest quarter, and precipitation seasonality. The bioclimatic variables were derived from WorldClim with resolution of ~1 km (http://www.worldclim.org/).

### 2.4 Spatial factors

Distance‐based Moran Eigenvectors maps, formerly called principal coordinates of neighbor matrices (PCNM; Borcard & Legendre, 2002) analysis based on overland distances among sites was first used to create spatial variables. This method can be applied to any set of sites providing a good coverage of the geographic sampling area. The PCNM represents the spatial configuration of sample units using principal coordinates of a truncated (nearest neighbors only) among samples distance matrix. From all the PCNMs, we retained those associated with significant Moran's I and positive eigenvalues in the subsequent analyses because they represent positive spatial autocorrelation (Gilbert & Bennett, 2010). PCNMs with small eigenvalues (e.g., PCNM1) represent large‐scale geographical/spatial patterns and large eigenvalues (here, e.g., PCNM26) represent small‐scale geographical/spatial patterns in of biological communities and univariate metrics values (in our case). We used the vegan's “pcnm” function in R (R Core Team, 2016) to generate the spatial PCNM axes for this analysis, and retained 26 PCNM axes (PCNM1‐PCNM26) with positive eigenvalues.

It seems that dispersal via stream channels is also important, especially in the dendritic stream networks (Seymour, Fronhofer, & Altermatt, 2015). However, in larger spatial scales across several catchments with relatively weak connectivity of stream channels (such as our study area), dispersal through watercourse should not be the major way of colonization for benthic animals. In contrast, overland dispersal over long distances within and across catchments may be common, most likely by means of passive dispersal modes or because the distance between the two adjacent headwaters is within the dispersal range of some flying adults (Li, Sundermann, Stoll, & Haase, 2016). In view of this, we only use spatial factors based on overland distance to detect spatial effects on biodiversity patterns in the current analysis. We here did not consider “region” (i.e., sub‐basin) as a large‐scale spatial factor constraining species distributions, because the effects of “region” per se on biodiversity patterns should be identical with the landscape‐climate factors (i.e., the basin characteristics:altitude, temperature, and precipitation) used in the analysis.

### 2.5 Measures of biodiversity

First, we calculated species richness, Shannon–wiener diversity and evenness index for each sampling site to represent taxonomic alpha diversity.

Next, thirteen traits belonging to four trait groups (Life history, Mobility, Morphology, and Ecology) for macroinvertebrates were selected and then subsequently divided into a total of 41 categories (Table 1). Although there are many other traits that would be useful in characterizing macroinvertebrate trait niches, the traits included here have previously been commonly adopted as indirect indicators of freshwater ecosystem functions (Lecerf et al., 2006; Usseglio‐Polatera, Bournaud, Richoux, & Tachet, 2000), and are thus suitable for the purposes of this study. We obtained trait information mainly from published articles and literatures (Poff et al., 2006; Usseglio‐Polatera et al., 2000; Vieira et al., 2006). We used the “dbFD” function in the FD package (Laliberté & Legendre, 2010) based on the relative abundance of taxa in each trait category to calculate functional richness (FRic), functional evenness (FEve), functional divergence (FDiv), and Rao's Quadratic Entropy (RaoQ). FRic, FEve, and FDiv are recognized as the primary components of functional diversity, and used to measure the overall spread of traits, and the evenness and divergence of abundance spread in trait space, respectively (Mason et al., 2005; Villéger et al., 2008). RaoQ measures the distribution of traits and abundance in trait space simultaneously, making it a useful index in explaining the relationships between assembly processes and environmental constraints (Rao, 1982).

**Table 1:** Functional trait classification of benthic macroinvertebrates in Tibetan streams

Trait groups	Trait	Trait state	Code
Life history	Voltinism	Semivoltine	Vol1
		Univoltine	Vol2
		Bi‐ or multivoltine	Vol3
	Development	Fast seasonal	Dev1
		Slow seasonal	Dev2
		Nonseasonal	Dev3
Mobility	Adult flying strength	Weak (e.g., cannot fly into light breeze)	Flgt1
		Strong	Flgt2
	Occurrence in drift	Rare (catastrophic only)	Drif1
		Common (typically observed)	Drif2
		Abundant (dominant in drift samples)	Drif3
	Swimming ability	None	Swim1
		Weak	Swim2
		Strong	Swim3
Morphology	Armoring	None (soft‐bodied forms)	Arm1
		Poor (heavily sclerotized)	Arm2
		Good (e.g., some cased caddisflies)	Arm3
	Size at maturity	Small (<9 mm)	Size1
		Medium (9–16 mm)	Size2
		Large (>16 mm)	Size3
	Shape	Streamlined (flat, fusiform)	Shp1
		Not streamlined (cylindrical, round, or bluff)	Shp2
	Respiration	Respiration Tegument	Res1
		Gills	Res2
		Valve, trachea, gas film	Res3
Ecology	Rheophily	Depositional only	Rhe1
		Depositional and erosional	Rhe2
		Erosional	Rhe3
	Thermal preference	Cold stenothermal or cool eurythermal	Ther1
		Cool/warm eurythermal	Ther2
		Warm eurythermal	Ther3
	Habit	Burrow	Hab1
		Climb	Hab2
		Sprawl	Hab3
		Cling	Hab4
		Swim	Hab5
		Skate	Hab6
	Trophic groups	Collector‐gatherer	Tro1
		Collector‐filterer	Tro2
		Herbivore (scraper, piercer, and shedder)	Tro3
		Predator (piercer and engulfer)	Tro4
		Shredder (detritivore)	Tro5

Third, we used taxonomic distinctness indices as a proxy for phylogenetic diversity, as we currently do not have true phylogeny comprising all the species in our data. This approach has been used in many studies dealing with phylogenetic diversity (Heino & Tolonen, 2017b; Tolonen et al., 2017; Warwick & Clarke, 1995). We thus calculated average taxonomic distinctness (AvTD; Warwick & Clarke, 1995) and variation in taxonomic distinctness (VarTD; Clarke & Warwick, 2001) for each site based on six taxonomic levels: species, genus, family, order, class, and phylum. AvTD index measures the mean taxonomic (or phylogenetic) distance between any two species in an assemblage, while VarTD index indicates the variance of these pairwise path lengths and reflects the unevenness of the taxonomic tree (Clarke & Warwick, 2001). Taxonomic diversity indices, AvTD and VarTD were calculated using PRIMER version 6 (Gibson, Barnes, & Atkinson, 2001).

### 2.6 Data analysis

Non‐normal response variables (biodiversity indices) and environmental variables, except pH, were transformed (arcsine‐square root for proportional data and log_10_ for continuous data) to improve their normality before statistical analysis. Pearson correlation analysis was used to test for the congruence between the biodiversity indices both in the whole study area and within each sub‐basin. Furthermore, we checked for the spatial autocorrelation in the response variables (the seven alpha diversity indices) based on Moran I index using the function “moran.test” implemented in the spdep R package. These background analyses showed that there was at best low spatial autocorrelation in the response variables (Moran I < 0.1, Supporting Information Table S1).

Multiple linear regression models (MLR) were used to examine the relationships between diversity indices and each set of explanatory variables (Preacher, Curran, & Bauer, 2006). This simple and heuristic approach is widely used to determine the ecological drivers most strongly associated with biodiversity patterns (Cai, Zhang, Xu, & Heino, 2018; Heino & Tolonen, 2017b). Prior to the analyses, highly correlated independent variables (Pearson's *r* > 0.75) from each set of environmental variables (i.e., landscape‐climate and habitat‐scale variables) were removed to reduce multicollinearity. This cross‐correlation analysis retained 6 landscape and 13 habitat‐scale variables (Table 2), and these variables along with the retained 26 spatial factors were then used in the subsequent statistical analyses.

**Table 2:** Descriptive statistics of environmental variables and biodiversity indices of macroinvertebrate assemblages across the 55 stream sites. “Discard” refers to the variables that were excluded from the analyses because of high collinearity with other variables

Variable	Abbreviation	Mean	*SD*	Min	Max	Discard
Habitat scale						
Water temperature (°C)	WT	10.44	3.53	3.3	16.7	NO
Conductivity (mS/cm)	EC	83.14	67.36	13.1	302.6	NO
Dissolved oxygen (mg/L)	DO	8.86	1.04	6.15	11.51	NO
pH	pH	8.31	0.45	7.14	8.8	NO
Channel width (m)	Wid	28.66	21.99	1	70	NO
Current velocity (ms^−1^)	Vel	1.02	0.41	0.1	1.88	NO
Mean depth (m)	MD	0.25	0.08	0.1	0.5	NO
Coarse woody debris	CWD	0.6	0.74	0	2	NO
Percentage of Boulder substrate	% Boulder	0.24	0.14	0	0.55	NO
Percentage of Cobble substrate	% Cobble	0.29	0.11	0	0.45	NO
Percentage of Pebble substrate	% Pebble	0.22	0.1	0	0.5	NO
Percentage of Gravel substrate	% Gravel	0.12	0.07	0	0.35	NO
Percentage of Sand substrate	% Sand	0.13	0.19	0	0.9	NO
Landscape scale						
Altitude	Alti	2224.22	1174.38	591.5	4563	YES
Annual mean temperature	ATem	7.53	4.88	−2.4	15.7	YES
Isothermality (2/7) (×100)	Iso	44.48	0.55	43.5	46	NO
Temperature seasonality (STD × 100)	TSea	581.91	50.59	519.6	712.5	YES
Max temperature of warmest month	TMax	19.89	4.04	10.3	27.1	NO
Min temperature of coldest month	TMin	−8.32	5.98	−21.8	0.8	NO
Temperature annual range	TRan	28.21	2.51	24.9	34.1	NO
Annual precipitation	APre	939.53	486.3	285	1785	NO
Precipitation of wettest month	PreWM	204.64	102.31	80	384	YES
Precipitation of driest month	PreDM	4.18	2.72	0	9	YES
Precipitation seasonality	PreSea	99.21	8.08	90.3	127.7	NO
Diversity indices						
Species richness	SRic	16.71	5.81	6	29	
Shannon–Wiener	Shan	2.13	0.07	2.49	1.75	
Evenness	Even	0.76	0.21	0.98	0.7	
Functional richness	FRic	64.57	42.13	0.24	165.21	
Functional divergence	FDiv	0.62	0.11	0.33	0.86	
Functional evenness	FEve	0.83	0.1	0.53	0.96	
Rao's Quadratic entropy	RaoQ	16.29	3.98	6.57	27.56	
Average taxonomic distinctness	AvTD	61.2	2.08	51.85	64.35	
Variation in taxonomic distinctness	VarTD	176.73	110.63	48.65	632.07	

Using MLR analysis, we first tested if the global test of the regression model was significant (Blanchet, Legendre, & Borcard, 2008). If the global model was not significant, no further analyses were conducted and we concluded that environmental (landscape‐climate or habitat‐scale) or spatial variables were not significant for the index considered. If the global model was significant, a forward selection using the function “ordiR2step” in vegan was conducted on each set of environmental and spatial (PCNMs) variables separately to select variables with significant contribution (*p* < 0.05, after 999 random permutations) to explaining variation for each response variable (Oksanen et al., 2013). Forward selection was conducted with two stopping rules: the adjusted *R* ^2^ value of the reduced model exceeded that of the global model or the critical *p* value (*p* = 0.05) was exceeded (Blanchet et al., 2008). Alternatively, we also considered using second‐order terms in the regression analyses. However, the regression models based on second‐order terms were either nonsignificant or had lower adjusted *R* ^2^‐values than the models we finally selected here.

To examine the relative importance of environmental variables and spatial factors explaining variation in each diversity index, variation partitioning based on partial linear regression was performed using the “varpart” function (Oksanen et al., 2013). The total percentage of variation explained divided into a unique and shared contribution for three sets of predictors using variation partitioning: (a) habitat variables; (b) landscape‐climate variables; and (c) PCNMs spatial vectors. We report adjusted *R* ^2^ (Adj. *R* ^2^) of pure and shared contributions of the spatial and environmental factors from the constrained ordinations, because of their impartiality and high recommendation in previous studies (Kromrey & Hines, 1995). We also tested for the significance of pure fractions of each set of predictor variables using the function “ANOVA” in the package vegan. Pearson correlation, MLR, and variation partitioning analyses were all conducted in R‐language environment (R Core eam, 2016).

## 3 RESULTS

**Figure 1:**
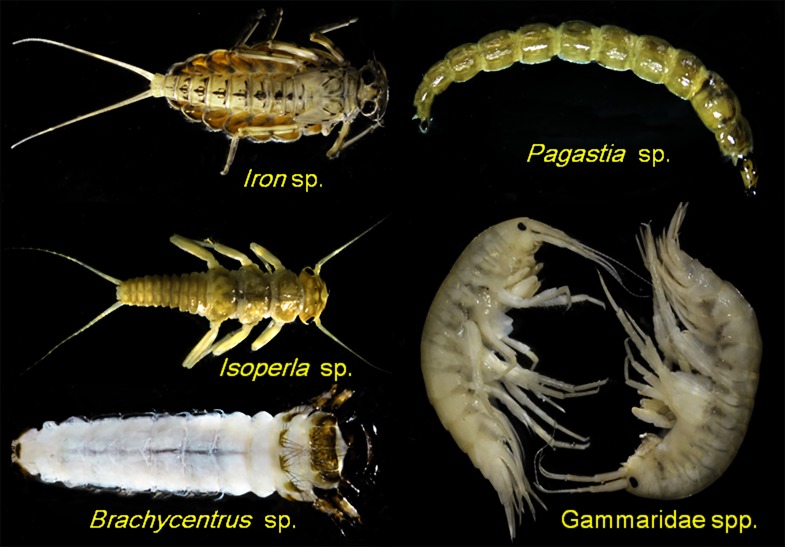
Some common benthic invertebrates in streams of southeastern Tibet

A total of 195 taxa were identified, belonging to 4 phyla, 6 classes and 66 families (Figure 1; Supporting Information Table S2). Aquatic insects contributed 94% of the total richness, with Ephemeroptera (45.9% relative abundance, 21 taxa overall), Chironomidae (17.2%, 63), Trichoptera (16.5%, 34), and Plecoptera (7.1%, 20) being the taxonomically richest groups. Baetidae (*Baetis* sp. and *Baetiella* sp.), Heptageniidae (*Iron* sp. and *Epeorus* sp.), and Chironomid (*Micropsetra *sp.) were the most common and abundant families in the study streams.

**Figure 2:**
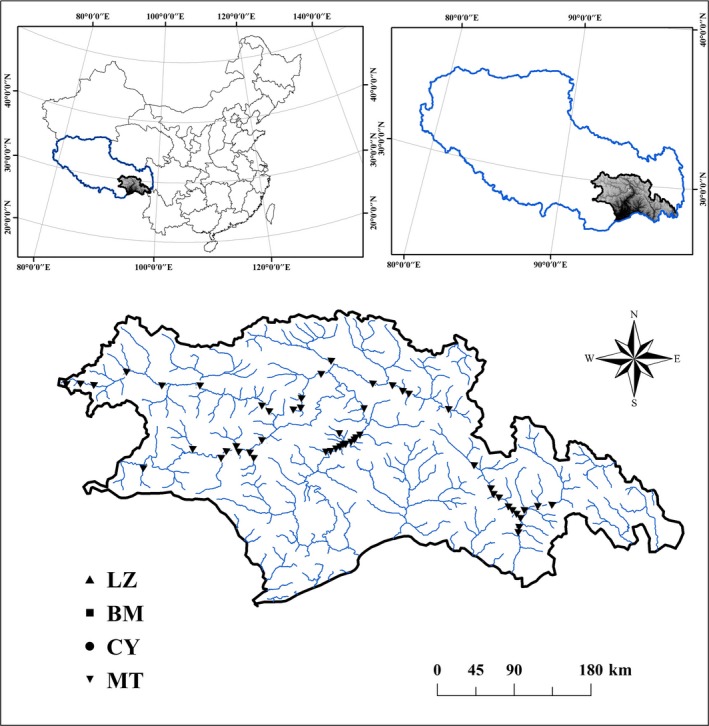
Geographical locations of the 55 study sites in southeastern Tibet, China

**Figure 3:**
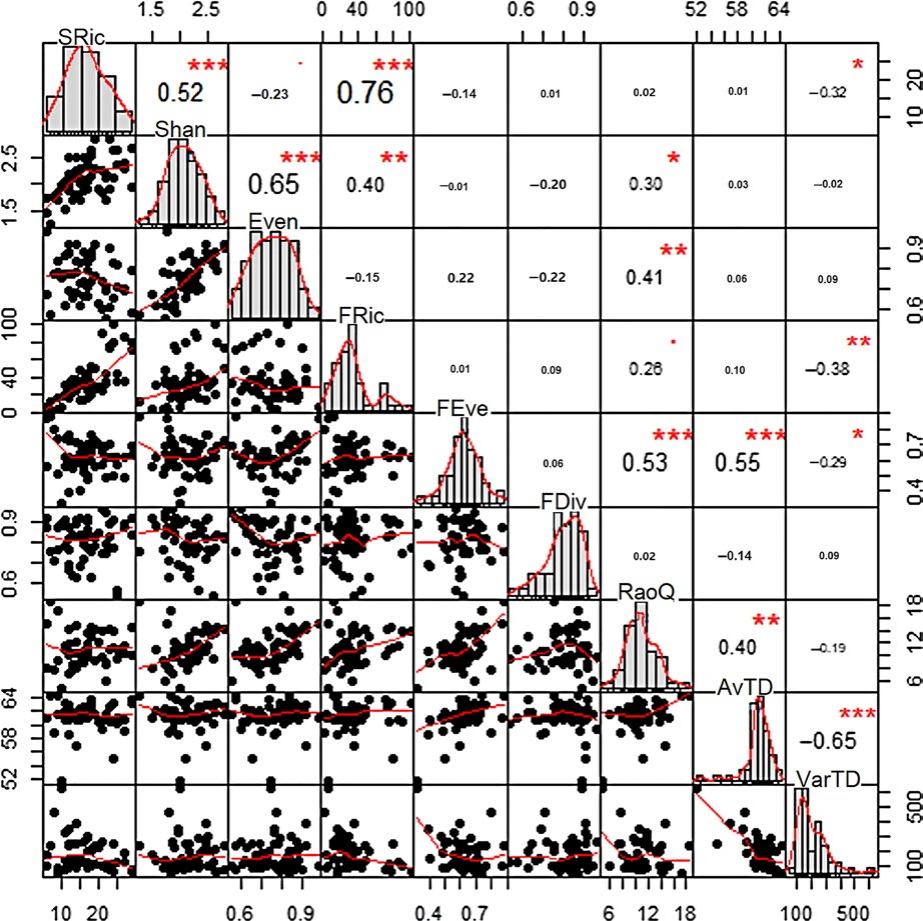
Scatter plots of correlations between different biodiversity indices. Pearson correlation coefficients are shown in the upper diagonal. *p < 0.05, **p < 0.01, ***p < 0.001

The biodiversity indices showed different degrees of variation across the sampling sites (Table 2). In the pairwise comparison between each pair of biodiversity indices, the correlations between the biodiversity indices were variable. In the whole study area, SRic were found correlated significantly with FRic (*r* = 0.76, *p* < 0.001) and Shannon diversity (*r* = 0.52, *p* < 0.00), while others were quite independent from SRic (Figure 3). Further, as a composite index incorporating both richness and evenness, Shannon diversity also correlated positively with evenness index (*r* = 0.65, *p* < 0.001). FEve index showed a significant correlation (*r* = 0.53, *p* < 0.001) with RaoQ, and both of the two indices were positively correlated with phylogenetic diversity indices (i.e., AvTD and VarTD). Further, AvTD and VarTD were strongly and negatively correlated. When analyses were conducted for each sub‐basin separately (Supporting Information Figure S1), the case was somewhat like the ones in the whole study area. For example, the consistent close relationships between FRic versus SRic, Shannon diversity versus SRic and evenness, AvTD versus VarTD, and the almost irrelevant connection between phylogenetic diversity and taxonomic diversity. However, the strength of correlations between biodiversity indices varied among study basins (Supporting Information Table S3), implying a context dependent of the relationships of diversity indices.

Based on the global models of MLR analysis, the data sets of habitat‐scale variables and spatial vectors were found significant for all of the biodiversity indices involved, while the landscape‐climate variables were found significant only for SRic and FRic. According to the forward selection procedure, the number and identity of environmental variables and spatial vectors explaining significant variation in biodiversity varied among the different facets of alpha diversity and diversity indices within a certain facet (Table 3).

**Table 3:** Results of multiple regression analyses. Separate analyses were run for taxonomic diversity indices (SRic, Shannon diversity and evenness), functional diversity indices (FRic, FEve, FDiv and RaoQ) and phylogenetic diversity indices (AvTD and VarTD). Adj. R^2^ values are in bold

Indices	Variables	Estimate	SE	t	*p*	Adj. R^2^
**SRic**	Habitat					
	(Intercept)	−14.221	12.83	−1.108	0.273	
	CWD	4.809	0.832	5.779	<0.001	
	DO	12.288	5.615	2.188	0.033	
						**0.402**
	Landscape					
	(Intercept)	12.057	1.883	6.403	< 0.001	
	APre	2.4753	0.921	2.688	0.01	
						**0.103**
	Spatial					
	(Intercept)	16.709	0.701	23.849	<0.001	
	PCNM3	−12.425	5.196	−2.391	0.021	
	PCNM23	−16.344	5.196	−3.146	0.003	
						**0.201**
**Shan**	Habitat					
	(Intercept)	2.40839	0.15659	15.38	<0.001	
	Wid	−0.125	0.043	−2.879	0.006	
	CWD	0.161	0.05975	2.694	0.009	
						**0.367**
	Landscape					
	**–**					
	Spatial					
	(Intercept)	2.131	0.045	47.552	<0.001	
	PCNM6	−0.516	0.332	−2.553	0.014	
	PCNM2	0.596	0.332	2.796	0.008	
						**0.130**
**Even**	Habitat					
	(Intercept)	0.754	0.021	18.889	<0.001	
	CWD	0.169	0.058	2.923	0.005	
	Wid	−0.028	0.012	−2.216	0.031	
						**0.210**
	Landscape					
	–					
	Spatial					
	(Intercept)	0.756	0.012	61.033	<0.001	
	PCNM3	0.265	0.092	2.882	0.006	
	PCNM2	0.236	0.091	2.572	0.013	
						**0.193**
**FRic**	Habitat					
	(Intercept)	−22.431	17.923	−1.252	0.216	
	WT	19.495	7.318	2.664	0.01	
	CWD	18.249	3.428	5.324	<0.001	
						**0.366**
	Landscape					
	(Intercept)	15.939	7.474	2.133	0.038	
	APre	10.138	3.655	2.774	0.008	
						**0.105**
	Spatial					
	(Intercept)	34.992	2.976	11.759	<0.001	
	PCNM22	56.254	22.068	2.549	0.014	
						**0.113**
**FEve**	Habitat					
	(Intercept)	0.724	0.036	20.154	<0.001	
	% Gravel	−0.528	0.17	−3.107	0.003	
						**0.138**
	Landscape					
	–					
	Spatial					
	(Intercept)	0.838	0.034	24.322	<0.001	
	PCNM23	0.03	0.015	2.012	0.049	
						**0.050**
**FDiv**	Habitat					
	(Intercept)	0.499	0.099	5.042	<0.001	
	WT	0.098	0.036	2.692	0.01	
	pH	0.339	0.148	2.296	0.026	
						**0.139**
	Landscape					
	**–**					
	Spatial					
	(Intercept)	0.811	0.012	65.604	< 0.001	
	PCNM17	0.205	0.092	2.231	0.03	
	PCNM10	−0.198	0.092	−2.159	0.036	
						**0.124**
**RaoQ**	Habitat					
	(Intercept)	14.495	1.098	13.201	<0.001	
	Vel	−5.038	1.54	−3.271	0.002	
						**0.152**
	Landscape					
	–					
	Spatial					
	(Intercept)	11.072	0.338	32.768	<0.001	
	PCNM23	7.274	2.505	2.903	0.005	
						
						**0.121**
**AvTD**	Habitat					
	(Intercept)	62.983	0.853	73.845	<0.001	
	% Sand	−0.597	0.271	−2.208	0.032	
						**0.067**
	Landscape					
	**–**					
	Spatial					
	(Intercept)	61.197	0.239	255.602	<0.001	
	PCNM6	5.551	1.776	3.126	0.003	
	PCNM7	5.089	1.776	2.866	0.006	
	PCNM23	3.989	1.775	2.247	0.029	
						**0.271**
**VarTD**	Habitat					
	(Intercept)	329.158	109.6	3.003	0.004	
	EC	−117.878	48.328	−3.105	0.003	
						**0.138**
	Landscape					
	**–**					
	Spatial					
	(Intercept)	98.377	4.824	20.394	< 0.001	
	PCNM23	−123.41	35.773	−3.45	0.001	
	PCNM10	−103.211	35.773	−2.885	0.006	
						**0.252**

**Figure 4:**
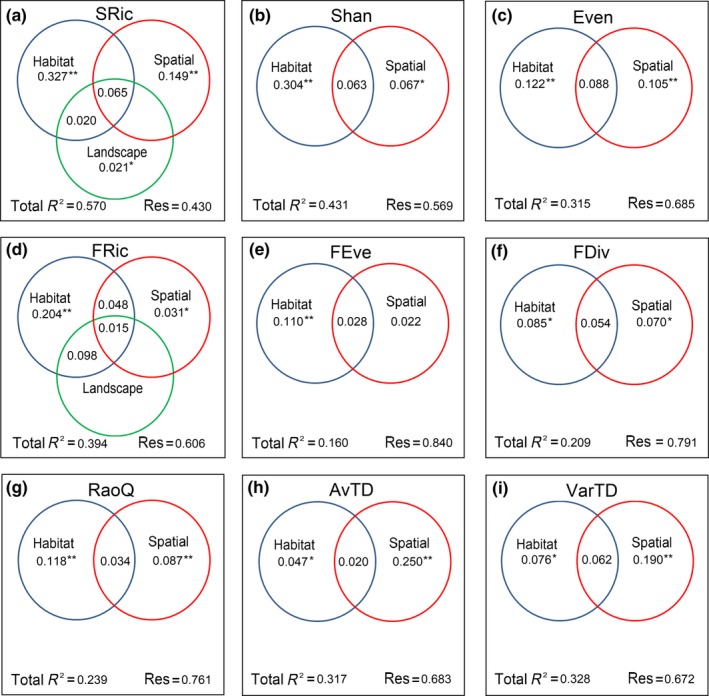
The relative contribution of habitat variables, landscape‐climate variables (landscape), and PCNM eigenvectors (spatial) to species richness (a), Shannon diversity (b), Evenness (c), FRic (d), FEve (e), FDiv (f), RaoQ (g), AvTD (h), and VarTD (i). Values represent the adjusted R2‐values. *p < 0.05, **p < 0.01. Negative fraction values are not presented

For species richness, forward selection identified two significant habitat variables (CWD and DO), one landscape‐climate variable (APre) and two spatial factors (PCNM 3 and PCNM 23) (Table 3.). A total of 57% of the variation in species richness could be explained by the three set of predictors based on the adjusted *R* ^2^‐values. Habitat variables alone explained more of the variance (32.7%) compared to the landscape‐climate (2.1%) and spatial parameters (14.9%, Figure 4a). Almost the same environmental variables (CWD and Wid) and broad‐scale spatial factors (PCNM 2 and PCNM 6 or PCNM 3) were selected for Shannon diversity and evenness index. And the variations in the two indices were more explained by habitat variables (30.4% for Shannon and 12.2% for evenness) than spatial factors (6.4% for Shannon and 10.5% for evenness, Figure 4b,c).

For FRic, two habitat variables (WT and CWD), one landscape variable (APre) and one spatial vector (PCNM22) were retained (Table 3). However, after variation partitioning, pure landscape effect was found to be nonsignificant (Figure 4d). Habitat variables accounted for a greater proportion of the variance in FRic (20.4%) compared to spatial vectors (3.1%). Only one habitat variable (% Gravel) and one spatial vector (PCNM23) were recorded to significantly influence FEve and accounted for 11% and 2.2% of the variance recorded, respectively (Figure 4e). Two habitat variables (WT and MD) and two spatial vectors (PCNM17 and PCNM10) significantly influenced FDiv and accounted for 8.5% and 7.0% of the total variance. For RaoQ, one habitat variable (Vel) and one spatial vector (PCNM7) were selected, and habitat variable still played more important roles (11.8%) than spatial vector (8.7%).

For phylogenetic alpha diversity, three spatial vectors (PCNM6, PCNM7, and PCNM23) and one habitat variable (% Sand) were selected for AvTD, and the variation was more effectively explained by spatial vectors (25%) compared to habitat variable (4.7%; Figure 4h). For VarTD, two spatial vectors (PCNM23 and PCNM10) and one habitat variable (EC) were retained, and the variance was more significantly explained by spatial vectors (19%) than habitat variables (7.6%).

## 4 DISCUSSION

### 4.1 Relationships between different facets of biodiversity

Recently, ecological, biogeographical, and conservation studies have highlighted the importance of using multiple facets of diversity in biodiversity analysis and environmental assessment, as the different facets may be complementary and provide different information about various ecological processes (Corbelli et al., 2015; Heino & Tolonen, 2017b; Tolonen et al., 2017). In this study, we focused on examining the relationship between multiple facets of alpha diversity of stream macroinvertebrates and their ecological drivers (i.e., environmental and spatial) in the Yarlung Zangbo Grand Canyon area, a near‐pristine river basin in the world. We found that some biodiversity indices were correlated to a certain extent, while others showed no significant coherence (Figure 3, Supporting Information Table S3). In addition, the strength of the relationships between biodiversity facets was different among study basins (i.e., the whole study basin and each sub‐basin) and different diversity indices. This indicates, as was the case in this study, that the relationships between biodiversity indices in aquatic ecosystems may be highly variable, context dependent, and affected by the diversity measures involved (Pavoine, Gasc, Bonsall, & Mason, 2013; Tolonen et al., 2017).

Nevertheless, we indeed found some regular patterns about the similarity or dissimilarity among these biodiversity indices between the three diversity facets. FRic showed consistently strong and positive correlation with SRic, despite the dataset (the entire study area or within each sub‐basin) chosen for analyses (Figure 3, Supporting Information Table S3). This finding was in accordance with those in previous investigations (e.g., Villéger et al., 2008), as FRic reflects the range of the trait values and is always constrained by SRic (Mason et al., 2005; Villéger et al., 2008). Other functional diversity indices (i.e., FEve, FDiv, and RaoQ), however, showed either significant positive, significant negative or nonsignificant relationships with taxonomic diversity indices among different geographical scopes. In most of the cases, rather weak correlations were observed between phylogenetic diversity (AvTD and VarTD) and taxonomic diversity measures, supporting previous studies which revealed a weak relationship between taxonomic distinctness and traditional diversity indices (Jiang, Song, Xiong, & Xie, 2014). This finding indicated that the taxonomic distinctness indices may represent different dimension among diversity facets, and may provide additional information about biodiversity and ecosystem conditions (Tolonen et al., 2017). In some cases, functional diversity indices were found significantly correlated with phylogenetic diversity indices (Figure 3, Supporting Information Table S3). This finding partially supported the perspective that functional and phylogenetic diversity should be related to each other due to evolutionary conservatism (i.e., many ecologically relevant traits harbor some degree of phylogenetic signal) (Winter, Devictor, & Schweiger, 2013). Thus, conserving phylogenetic diversity may become an effective strategy for conserving functional diversity and partially ensure the maintenance of ecosystem function (Webb et al., 2002).

### 4.2 Key environmental and spatial factors driving different facets of biodiversity

The three facets of alpha diversity responded differently to each set of predictor variables, which was in line with a priori hypothesis. At the local scale, habitat variables regarding habitat heterogeneity (i.e., CWD, substrates, MD and Vel) and water physic‐chemistry attribute (i.e., DO, WT, pH and EC) played important roles for variation in the indices of the three facets. For the taxonomic facet, species richness, Shannon diversity, and evenness correlated positively with CWD values (Table 3), which basically matched the conclusion of previous studies who reported a strong relationship between CWD scores and macroinvertebrate taxonomic diversity in‐stream ecosystems (Arnaiz et al., 2011; Czarnecka, Pilotto, & Pusch, 2015; Milner & Gloyne‐Phillips, 2005). In addition, species richness was also strongly related to dissolved oxygen which was recognized as a driving factor for the decline in taxon richness of macroinvertebrates in high‐altitude streams, mainly by reducing the metabolism and bringing sublethal effects to these organisms (Jacobsen, 2008). Channel width was detected have a negative effect on Shannon diversity and evenness. In our study, channel width could serve as an integrated descriptor of the heterogeneity to a certain extent. The increasing channel width accompanied by decreasing CWD values and increasing diminishing substrate size can reduce habitat heterogeneity in sampling sites and decrease evenness and diversity of benthic assemblages ultimately.

Similar to the case of taxonomic diversity, habitat complexity and heterogeneity (CWD and substrates) played a primary role in determining functional diversity. Such findings supported the habitat template theory, which indicates that habitat features select species with suitable traits to coexist in a local community and filter out the ones without such traits (Southwood, 1977; Townsend & Hildrew, 1994). The increasing habitat heterogeneity provides more opportunities for niche partitioning and, in turn, are reflected by higher functional diversity (Stark, Lehman, Crawford, Enquist, & Blonder, 2017). These opportunities in streams should include the diversity of food resources for feeding (e.g., CWD) and different habitat structural characteristics for refugia from flood disturbance (substrates). In‐stream ecosystems, fine substrates have a uniformly negative influence on both richness and abundance of macroinvertebrate communities owing to the lack of adequate refuges with high heterogeneity and stability (Jiang et al., 2013; Reice, 1980). Such adverse conditions can also exert influence on trait composition and thus functional diversity, which may shed light in explaining the negative relationship between functional evenness and fine substrate (i.e., % Gravel). Apart from habitat complexity, functional diversity also correlated positively to habitat mildness (i.e., the increasing WT and pH). This finding may be related to the possibility that increasing habitat mildness allowed more species with different specialized niches to coexist and maintain viable populations in local communities, whereas harsh habitat conditions may indirectly reduce the versatility of traits and rate of ecosystem processes (Heino, 2005). Current velocity was also found to have a significant impact on the functional diversity of macroinvertebrate communities. Suitable current velocity conditions are a requirement for stream fauna, especially for the rheophile taxa. However, excessive current velocity (e.g., floods) can also serve as a strong filter, virtually washing away species indiscriminately, despite the suitability of specific traits for the local habitats (Gallardo, Gascón, García, & Comín, 2009; Townsend, Scarsbrook, & Dolédec, 1997). The unfavorable conditions limit the range of life‐history strategies capable of supporting survival which may explain the negative correlation between RaoQ and current velocity (Statzner, Dolédec, & Hugueny, 2004). The phylogenetic diversity indices (AvTD and VarTD) were negatively correlated with fine substrate (% Sand) and conductivity. Such findings indicated that the low habitat complexity and strong natural disturbance (as the high water conductivity occurred in the glacier‐fed streams) only allows the coexistence of those lineages with suitable traits to persist in harsh environments, known as habitat filtering (Anacker & Harrison, 2012). Although beyond the scope of our present study, we predict that these traits should be related to small body size, flexible mobility and multivoltinism, and these species should possibly be habitat generalists (Heino, Schmera, & Erős, 2013).

Only one landscape‐climate variable regarding precipitation (Annual Precipitation) in our study was detected significantly influenced species richness and functional richness (Table 3). This is not surprising because changes in precipitation regimes are likely to exert influence on rates and pathways of surface runoff (Wen, Lin, Xue, & Sun, 2010). This can result in geographic variation in‐stream flow regimes, which directly or indirectly regulate macroinvertebrate community structure and function (Poff et al., 2010).

Apart from environmental variables, spatial factors related to both large and fine scales strongly influenced patterns of the three biodiversity facets. Developing a comprehensive multiscale understanding of spatial patterns in community structure and biodiversity is a goal for ecologists, and the development of the PCNM method (Borcard & Legendre, 2002) is an important step forward in this process. PCNM uses metric geographic coordinates and takes into account complex spatial structures in biological communities, which may result from environmental autocorrelation, dispersal, historical effects, or other ecological processes (Dray et al., 2012). We found that PCNM vectors related to broad‐scale spatial patterns (e.g., PCNM2 & PCNM3) were identified as important predictors for taxonomic diversity. This may indicate an effect of dispersal limitation, or because some environmental factors describing broad‐scale species sorting or historical factors strongly influencing temporal species composition were reflected by these PCNM vectors. We found that functional diversity was typically correlated with PCNM vectors related to fine‐scale spatial patterns (e.g., PCNM22 & PCNM23). The detected spatial signals in functional diversity was thus consistent with results from other empirical studies (e.g., Heino, 2005; Colzani et al., 2013; Ding et al., 2017), implying that ecological opportunities and functional trait composition may also change along geographical gradients (Schmera, Erős, & Heino, 2013). The spatial structuring in functional diversity may result from several environmental variables acting at fine spatial scales (e.g., the ones describing among‐site habitat heterogeneity). The explanation of relationships between phylogenetic diversity and spatial factors should be more complicated, as both broad (e.g., PCNM6 and PCNM7) and fine‐scale (i.e., PCNM23) spatial vectors were selected. This finding partially imply the fact that phylogenetic diversity is related to multiple ecological processes, including those acting at broad scales (e.g., evolutionary and dispersal processes) and fine scales (i.e., biotic interactions and environmental filtering; Morlon et al., 2011).

### 4.3 Relative importance of environmental and spatial factors for different facets of biodiversity

The results based on variation partitioning suggested distinct driving mechanisms for different facets of biodiversity. Environmental variables at habitat scale played more important roles over landscape and spatial factors for both taxonomic and functional diversity. This was understandable because the streams in our study covered a wide range of environmental gradients in which the environmental variation or habitat harshness in these systems provided a large scope for environmental filtering (Liu et al., 2016). In view of the above‐mentioned facts, it was thus surprising that the variation of phylogenetic diversity was more strongly explained by spatial factors rather than environmental variables. This finding contrast with the perspective that phylogenetic data should be more influenced by deterministic process (environmental filtering) due to niche conservatism and trait–environment relationships (Poff, 1997; Webb et al., 2002).

We propose several possible causes that may explain such results. Firstly, dispersal limitation may be dominant in determining variation of phylogenetic diversity in such a metacommunity located in a relatively large geographical area, as the responses of phylogenetic diversity are more related to spatial scales (Heino & Tolonen, 2017a; Saito, Soininen, Fonseca‐Gessner, & Siqueira, 2015). Moreover, at still larger scales, such as the subtropical–temperate gradient in our study area, the distribution of phylogenetic diversity may be controlled by the species pool of each river basin (Anacker & Harrison, 2012). This can be seen as the absence of cold stenothermal lineages from the subtropical zones, which may account for the geographical variations in phylogenetic diversity in our study area. Secondly, the proxy for phylogenetic diversity, that is, distinctness indices are recorded sensitive to anthropogenic disturbances (Jiang et al., 2014; Tolonen et al., 2017), such as water quality and intensity of land use, which are almost nonexistent in our research basin. Finally, we believe that stronger relationships between phylogenetic diversity measures and environmental factors should have been detected if true phylogenetic information was used as the basis of the analyses.

Unlike the conclusions from other studies (Colzani et al., 2013; González‐Maya et al., 2016; Heino, Alahuhta, & Fattorini, 2015), landscape‐climate variables in our study were found to be almost negligible in explaining variation in biodiversity indices (except for SRic and FRic). Nonetheless, we cannot exclude the possibility that large‐scale environmental filtering effects are still important for biodiversity patterns, as local abiotic factors are constrained by landscape‐climate characteristics (Liu et al., 2016). The absence of landscape‐climate effects on biodiversity in our study may be because the large‐scale variables are more inclined to exert influence on biodiversity indirectly through interacting with local environmental variables (Colzani et al., 2013). Furthermore, some potentially important landscape variables for organisms in highland streams were not measured owing to lack of suitable background information, such as maps of topographic heterogeneity and vegetation coverage (Colzani et al., 2013; Knouft & Page, 2011).

The joint contribution of spatial vectors and environmental variables also explained a certain proportion of the variation in biodiversity patterns, although these shared effects were not more influential than their individual contributions (i.e., the pure effects). For stream macroinvertebrate assemblages, species in local communities have to pass filters at regional, watershed, channel unit, and microhabitat scales, each working at certain spatial scales (Poff et al., 2010). We argue that the shared effects should result from some environmental variables which were spatially structured. Furthermore, it should be noted that the amount of unexplained variation (from 43% to 84%) was relatively high in some models, which might be due to some unmeasured yet important factors. We thus argue that other sets of environmental variables as well as spatial factors should be considered for predicting biodiversity patterns in alpine streams, such as flow directionality and mountain barriers for spatial effects (Dong et al., 2016) and biotic interactions for environmental filtering (Colzani et al., 2013; Webb et al., 2002). Finally, we had to admit that a snapshot sampling of stream macroinvertebrate assemblages may not sufficiently reflect the strongest relationships between biodiversity patterns and ecological factors. However, our study indeed revealed that the three facets of biodiversity were determined by distinct ecological drivers which had basically reached the expected goal. We still argue that seasonal and interannual surveys of macroinvertebrate communities and biodiversity patterns should be necessary to assess the generality of the findings.

## 5 CONCLUSIONS

Generally, our results confirmed that the underlying environmental variables and spatial factors contributed differently to each facet of alpha diversity. Taxonomic and functional diversities were more strongly determined by environmental variables, while phylogenetic diversity showed stronger spatial structuring. Besides, environmental factors at the habitat scale played the dominant role over landscape‐climate variables in determining macroinvertebrate diversity patterns in our study. Such findings showed the complementary patterns of taxonomic, functional, and phylogenetic diversity, highlighting the importance of comprehensively considering multiple facets of diversity for efficient biodiversity assessment and conservation planning. Species distributions are driven by multiple evolutionary and ecological processes, including speciation and extinction, dispersal and environmental filtering, which occur at a variety of spatio‐temporal scales (Feng et al., 2014; Morlon et al., 2011). Such complexity makes it difficult to explain and predict biodiversity patterns which have become challenging tasks for conservation biogeography. Therefore, it is important to examine and predict changes in biodiversity patterns using environmental variables acting from landscape to microhabitat scales. Meanwhile, given the considerable degree of spatial structure in biodiversity patterns, the spatial context should also be explicitly considered in fundamental and applied research on stream biodiversity. More specifically, for biodiversity conservation in highland stream ecosystems, our results support the idea that using an integrative approach embracing multiple facets of diversity is essential.

## CONFLICT OF INTEREST

None declared.

## AUTHOR CONTRIBUTIONS

Zhengfei Li: Specimen collection and identification, data analysis and article writing; Zhicai Xie, Xiaoming Jiang, Jun Wang and Xingliang Meng helped in specimen collection and contributed ideas that led to the manuscript concept; Jani Heino: Providing valuable suggestions and comments for the manuscript. All authors contributed significantly to the writing of the paper.

## Data Availability

The data used in this manuscript were obtained from field investigation (habitat variables), laboratory experiment (taxon composition), published literature (functional traits, Usseglio‐Polatera et al., 2000; Poff et al., 2006; Vieira et al., 2006) and network resource (http://www.worldclim.org/, landscape‐climate variables). I have attached the taxon information in supplemental files, and I would like to submit the relevant raw data to in a public repository such as Dryad.
