# Supplementary material for: Multiple facets of stream macroinvertebrate alpha diversity are driven by different ecological factors across an extensive altitudinal gradient
Source: Ecol Evol. 2019 Jan 15;9(3):1306–22. doi: 10.1002/ece3.4841 (PMC6374682; doi:10.1002/ece3.4841)
Supplement: Supplementary file 1 [file ECE3-9-1306-s001.docx]

Table S1. Results of spatial autocorrelation analysis of the response variables (i.e. biodiversity indices).

|  | Moran's I | Expectation | Variance | *p*-value |
| --- | --- | --- | --- | --- |
| SRic | 0.033 | -0.019 | 0.0050 | 0.232 |
| FRic | -0.029 | -0.019 | 0.0049 | 0.558 |
| FEve | 0.038 | -0.019 | 0.0049 | 0.210 |
| FDiv | 0.105 | -0.019 | 0.0049 | 0.040 |
| RaoQ | 0.043 | -0.019 | 0.0049 | 0.189 |
| AvTD | 0.024 | -0.019 | 0.0042 | 0.257 |
| VarTD | 0.055 | -0.019 | 0.0048 | 0.147 |

Table S2. Taxon list of the identified 195 macroinvertebrate taxa in South Tibetan streams.

| **Species/Genus** | **Family** | **Order** |
| --- | --- | --- |
| *Ephemera* sp*.* | Ephemeridae | Ephemeroptera |
| *Baetis* sp. | Baetidae | Ephemeroptera |
| *Baetiella* sp. | Baetidae | Ephemeroptera |
| *Pseudocloeon* sp. | Baetidae | Ephemeroptera |
| *Drunella* sp. | Ephemerellidae | Ephemeroptera |
| *Serratella* sp. | Ephemerellidae | Ephemeroptera |
| *Cincticostella* sp. | Ephemerellidae | Ephemeroptera |
| *Ephacerella* sp. | Ephemerellidae | Ephemeroptera |
| *Ephemerella* sp | Ephemerellidae | Ephemeroptera |
| *Neoephemera* sp. | Neoephemeridae | Ephemeroptera |
| *Notacanthurus* sp. | Heptageniidae | Ephemeroptera |
| *Rhithrogena* sp. | Heptageniidae | Ephemeroptera |
| *Epeorus* sp. | Heptageniidae | Ephemeroptera |
| *Iron* sp. | Heptageniidae | Ephemeroptera |
| *Cinygmina* sp. | Heptageniidae | Ephemeroptera |
| *Heptagenia* sp*.* | Heptageniidae | Ephemeroptera |
| *Caenis* sp. | Caenidae | Ephemeroptera |
| *Siphlonurus* sp. | Siphlonuridae | Ephemeroptera |
| *Ameletus* sp. | Siphlonuridae | Ephemeroptera |
| *Habrophlebiodes* sp*.* | Leptophlebiidae | Ephemeroptera |
| *Leptophlebia* sp*.* | Leptophlebiidae | Ephemeroptera |
| *Hydropsyche* sp. | Hydropsychidae | Trichoptera |
| *Macrostemum* sp. | Hydropsychidae | Trichoptera |
| *Potamyia* sp. | Hydropsychidae | Trichoptera |
| *Cheumatopsyche* sp. | Hydropsychidae | Trichoptera |
| *Arctopsyche* sp. | Hydropsychidae | Trichoptera |
| *Anagapetus* sp. | Glossosomatidae | Trichoptera |
| *Agapetus* sp. | Glossosomatidae | Trichoptera |
| *Glossosoma* sp. | Glossosomatidae | Trichoptera |
| Brachycentrus sp. | Brachycentridae | Trichoptera |
| *Micrasema* sp. | Brachycentridae | Trichoptera |
| *Limnocentropus* sp. | Limnocentropodidae | Trichoptera |
| *Stenopsyche* sp. | Stenopsychidae | Trichoptera |
| *Apatania* sp. | Apataniidae | Trichoptera |
| *Polycentropus* sp. | Polycentropodidae | Trichoptera |
| *Neureclipsis* sp. | Polycentropodidae | Trichoptera |
| *Hydroptila* sp. | Hydroptilidae | Trichoptera |
| *Rhyacophila* sp1. | Rhyacophilidae | Trichoptera |
| *Rhyacophila* sp2. | Rhyacophilidae | Trichoptera |
| *Rhyacophila* sp3. | Rhyacophilidae | Trichoptera |
| *Rhyacophila* sp4. | Rhyacophilidae | Trichoptera |
| *Himalopsyche* sp1. | Rhyacophilidae | Trichoptera |
| *Himalopsyche* sp2. | Rhyacophilidae | Trichoptera |
| *Lepidostoma* sp1. | Lepidostomatidae | Trichoptera |
| *Lepidostoma* sp2. | Lepidostomatidae | Trichoptera |
| *Clostoeca* sp. | Limnephilidae | Trichoptera |
| *Glyphopsyche* sp. | Limnephilidae | Trichoptera |
| *Eocosmoecus* sp. | Limnephilidae | Trichoptera |
| *Homophylax* sp. | Limnephilidae | Trichoptera |
| *Limnephilus* sp. | Limnephilidae | Trichoptera |
| *Dolophilodes* sp. | Philopotamidae | Trichoptera |
| *Chimarra* sp. | Philopotamidae | Trichoptera |
| *Apsilochorema* sp. | Hydrobiosidae | Trichoptera |
| *Lype* sp. | Psychomyiidae | Trichoptera |
| Calamoceratidae spp. | Calamoceratidae | Trichoptera |
| *Mesonemura* sp. | Nemouridae | Plecoptera |
| *Amphinemura* sp. | Nemouridae | Plecoptera |
| *Nemoura* sp. | Nemouridae | Plecoptera |
| *Isoperla* sp. | Perlodidae | Plecoptera |
| *Stavsolus* sp. | Perlodidae | Plecoptera |
| *Helopicus* sp. | Perlodidae | Plecoptera |
| *Skwala* sp. | Perlodidae | Plecoptera |
| *Baumannella* sp. | Perlodidae | Plecoptera |
| *Capnia* sp. | Capniidae | Plecoptera |
| *Eucapnopsis* sp. | Capniidae | Plecoptera |
| *Paraleuctra* sp. | Leuctridae | Plecoptera |
| *Taenionema* sp. | Taeniopterygidae | Plecoptera |
| *Strophopteryx* sp. | Taeniopterygidae | Plecoptera |
| *Suwallia* sp. | Chloroperlidae | Plecoptera |
| *Cerconychia* sp. | Chloroperlidae | Plecoptera |
| *Peltoperlopsis* sp. | Peltoperlidae | Plecoptera |
| *Togoperla* sp. | Perlidae | Plecoptera |
| *Tetropina* sp. | Perlidae | Plecoptera |
| *Calineuria* sp. | Perlidae | Plecoptera |
| Perlidae spp. | Perlidae | Plecoptera |
| *Merogomphus* sp. | Gomphidae | Odonata |
| *Burmagomphus* sp. | Gomphidae | Odonata |
| *Styiogomphus* sp. | Gomphidae | Odonata |
| *Nepogomphus* sp. | Gomphidae | Odonata |
| Gomphidae spp. | Gomphidae | Odonata |
| *Macromia* sp. | Macromiidae | Odonata |
| *Cordulegaster* sp. | Cordulegastridae | Odonata |
| *Sympetrum* sp. | Libellulidae | Odonata |
| *Hetaerina* sp. | Calopterygidae | Odonata |
| *Dryops* sp. | Dryopidae | Coleoptera |
| *Berosus* sp. | Hydrophilidae | Coleoptera |
| *Dytiscus* sp. | Dytiscidae | Coleoptera |
| *Stenelmis* sp. | Elmididae | Coleoptera |
| *Optioservus* sp. | Elmididae | Coleoptera |
| *Gonielous* sp. | Elmididae | Coleoptera |
| Chrysomelidae spp. | Chrysomelidae | Coleoptera |
| *Aulacodes* sp. | Pyralidae | Lepidoptera |
| *Oxyelophila* sp. | Pyralidae | Lepidoptera |
| *Protohermes* sp. | Corydalidae | Megaloptera |
| Epiosmylidae spp. | Epiosmylidae | Neuroptera |
| *Micronecta* sp. | Corixidae | Hemiptera |
| *Antocha* sp. | Tipulidae | Diptera |
| *Hexatoma* sp1. | Tipulidae | Diptera |
| *Hexatoma* sp2. | Tipulidae | Diptera |
| *Epiphragma* sp. | Tipulidae | Diptera |
| *Pedicia* sp*.* | Tipulidae | Diptera |
| *Tipula* sp1. | Tipulidae | Diptera |
| *Tipula* sp2. | Tipulidae | Diptera |
| *Ilisia* sp. | Tipulidae | Diptera |
| *Dicranota* sp. | Tipulidae | Diptera |
| *Dactylolabis* sp. | Tipulidae | Diptera |
| *Simulium* sp. | Simulidae | Diptera |
| *Rhagionid* sp. | Rhagionidae | Diptera |
| *Tabanus* sp. | Tabanidae | Diptera |
| *Hemerodromia* sp. | Empididae | Diptera |
| *Oreogeton* sp. | Empididae | Diptera |
| Blephariceridae spp. | Blephariceridae | Diptera |
| Deuterophlebiidae spp. | Deuterophlebiidae | Diptera |
| *Dasyhelea* sp. | Psychodidae | Diptera |
| *Chironomaptera* sp. | Chaoboridae | Diptera |
| *Culicoides* sp. | Ceratopogonidae | Diptera |
| *Bezzia* sp. | Ceratopogonidae | Diptera |
| *Mallochohelea* sp. | Ceratopogonidae | Diptera |
| *Lucilia* sp. | Muscidae | Diptera |
| *Psilopa* sp. | Ephydridae | Diptera |
| *Demicryptochironomus* sp. | Chironominae | Diptera |
| *Microtendipes* sp. | Chironominae | Diptera |
| *Paratendipes* sp. | Chironominae | Diptera |
| *Polypedilum* sp. | Chironominae | Diptera |
| *Cryptochironomus* sp. | Chironominae | Diptera |
| *Stictochironomus* sp. | Chironominae | Diptera |
| *Parachironomus* sp. | Chironominae | Diptera |
| *Saethria* sp. | Chironominae | Diptera |
| *Chironomus* sp. | Chironominae | Diptera |
| *Apedilum* sp. | Chironominae | Diptera |
| Chironominae spp1. | Chironominae | Diptera |
| *Micropsetra* sp1. | Chironominae | Diptera |
| *Micropsetra* sp2. | Chironominae | Diptera |
| *Tanytarsus* sp1. | Chironominae | Diptera |
| *Tanytarsus* sp2. | Chironominae | Diptera |
| *Dicrotendipes* sp. | Chironominae | Diptera |
| *Cladotanytarsus* sp. | Chironominae | Diptera |
| *Rheotanytarsus* sp. | Chironominae | Diptera |
| *Paratanytarsus* sp. | Chironominae | Diptera |
| *Neozavrelia* sp. | Chironominae | Diptera |
| *Rheopelopia* sp. | Tanypodinae | Diptera |
| *Macropelopia* sp. | Tanypodinae | Diptera |
| *Conchapelopia* sp. | Tanypodinae | Diptera |
| *Ablabesmyia* sp. | Tanypodinae | Diptera |
| *Natarsia* sp. | Tanypodinae | Diptera |
| *Procladius* sp. | Tanypodinae | Diptera |
| *Tanypus* sp. | Tanypodinae | Diptera |
| *Paracladius* sp. | Orthocladiinae | Diptera |
| *Krenosmittia* sp. | Orthocladiinae | Diptera |
| *Orthocladius* sp1. | Orthocladiinae | Diptera |
| *Orthocladius* sp2. | Orthocladiinae | Diptera |
| *Cricotopus* sp1. | Orthocladiinae | Diptera |
| *Cricotopus* sp2. | Orthocladiinae | Diptera |
| *Corynoneura* sp. | Orthocladiinae | Diptera |
| *Aericotopus* sp. | Orthocladiinae | Diptera |
| *Paracricotopus* sp. | Orthocladiinae | Diptera |
| *Pseudorthocladius* sp. | Orthocladiinae | Diptera |
| *Epoicocladius* sp. | Orthocladiinae | Diptera |
| *Eukiefferiella* sp. | Orthocladiinae | Diptera |
| *Parakiefferiella* sp. | Orthocladiinae | Diptera |
| *Paratrichocladius sp.* | Orthocladiinae | Diptera |
| *Parametrioremns* sp. | Orthocladiinae | Diptera |
| *Cardiocladius* sp. | Orthocladiinae | Diptera |
| *Chaetocladius* sp. | Orthocladiinae | Diptera |
| *Psectrocladius* sp. | Orthocladiinae | Diptera |
| *Heleniella* sp. | Orthocladiinae | Diptera |
| *Heterotrissocladius* sp. | Orthocladiinae | Diptera |
| *Tveteniadiscoloripes* sp. | Orthocladiinae | Diptera |
| *Paracladius* sp. | Orthocladiinae | Diptera |
| *Rheocricotopus* sp. | Orthocladiinae | Diptera |
| *Brillia* sp. | Orthocladiinae | Diptera |
| *Limnophyes* sp. | Orthocladiinae | Diptera |
| *Nanocladius* sp. | Orthocladiinae | Diptera |
| *Thienemanniella* sp. | Orthocladiinae | Diptera |
| *Euryhapsis* sp. | Orthocladiinae | Diptera |
| *Compterosmittia* sp. | Orthocladiinae | Diptera |
| *Georthocladius* sp. | Orthocladiinae | Diptera |
| *Monodimesa* sp. | Diamesinae | Diptera |
| *Boreoheptagyia* sp. | Diamesinae | Diptera |
| *Pagastia* sp. | Diamesinae | Diptera |
| *Diamesa* sp. | Diamesinae | Diptera |
| *Potthastia* sp. | Diamesinae | Diptera |
| *Pseudodiamesa* sp. | Diamesinae | Diptera |
| Gammaridae spp. | Gammaridae | Amphipoda |
| *Grandidierella* sp. | Aoridae | Amphipoda |
| *Limnodrilus hoffmeisteri* | Tubificidae | Tubificida |
| *Limnodrilus* sp. | Tubificidae | Tubificida |
| *Telmatodrilus* sp. | Tubificidae | Tubificida |
| *Tubifex* sp. | Tubificidae | Tubificida |
| *Lumbriculus variegatum* | Tubificidae | Tubificida |
| *Pheretima* sp. | Megascolecidae | Haplotaxida |
| *Fridericia* sp. | Enchytraeidae | Tubificida |
| Hydracarina spp. | Hydracarina.fam | Acariformes |
| Turbellaria | Tricladida | Tricladida |
| Nematodes | Nematodes | Nematodes |


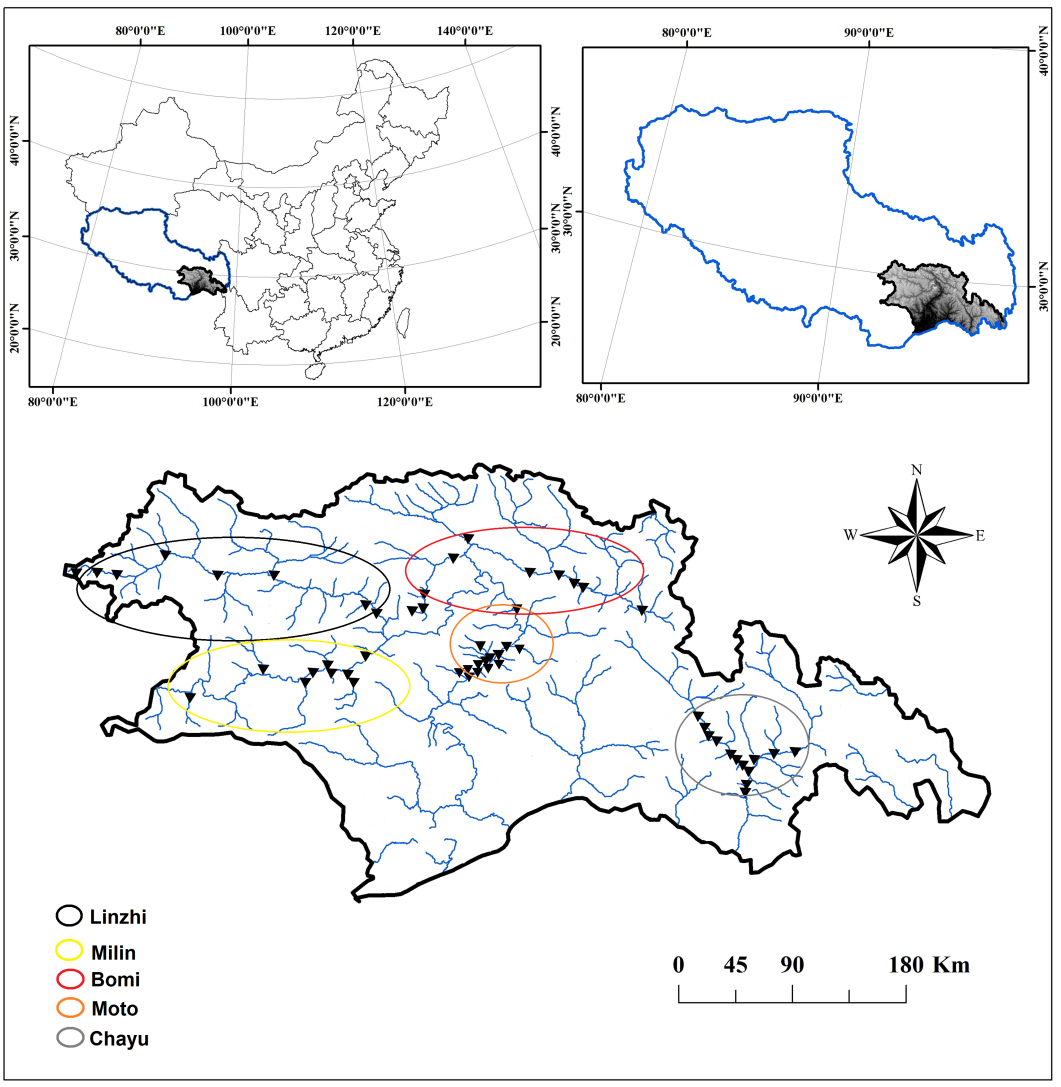


Figure S1 Geographical location of the 55 study sites distributed in 5 sub-basins of the Yarlung Zangbo River Basin, southeastern Tibet, China.

Table S3 Relationships (Pearson correlations) between biodiversity indices in 5 sub-basins of the Yarlung Zangbo River Basin.

|  |  | SRic | Shannon | Evenness | FRic | FEve | FDiv | RaoQ | AvTD | VarTD |
| --- | --- | --- | --- | --- | --- | --- | --- | --- | --- | --- |
| Linzhi | SRic |  |  |  |  |  |  |  |  |  |
|  | Shannon | 0.52 |  |  |  |  |  |  |  |  |
|  | Evenness | -0.23 | 0.65 |  |  |  |  |  |  |  |
|  | FRic | 0.76 | 0.40 | -0.15 |  |  |  |  |  |  |
|  | FEve | -0.14 | -0.01 | 0.22 | 0.01 |  |  |  |  |  |
|  | FDiv | 0.01 | -0.20 | -0.22 | 0.09 | 0.06 |  |  |  |  |
|  | RaoQ | 0.02 | 0.30 | 0.41 | 0.26 | 0.53 | 0.02 |  |  |  |
|  | AvTD | 0.01 | 0.03 | 0.06 | 0.10 | 0.55 | -0.14 | 0.40 |  |  |
|  | VarTD | -0.32 | -0.02 | 0.09 | -0.38 | -0.29 | 0.09 | -0.19 | -0.65 |  |
| Milin | SRic |  |  |  |  |  |  |  |  |  |
|  | Shannon | 0.53 |  |  |  |  |  |  |  |  |
|  | Evenness | -0.07 | 0.80 |  |  |  |  |  |  |  |
|  | FRic | 0.39 | 0.12 | -0.03 |  |  |  |  |  |  |
|  | FEve | -0.08 | 0.22 | 0.36 | 0.31 |  |  |  |  |  |
|  | FDiv | -0.16 | -0.48 | -0.51 | -0.65 | -0.28 |  |  |  |  |
|  | RaoQ | 0.11 | 0.37 | 0.46 | 0.61 | 0.80 | -0.41 |  |  |  |
|  | AvTD | 0.13 | 0.15 | 0.17 | 0.63 | 0.86 | -0.45 | 0.84 |  |  |
|  | VarTD | -0.35 | 0.03 | 0.18 | -0.92 | -0.49 | 0.51 | -0.64 | -0.82 |  |
| Bomi | SRic |  |  |  |  |  |  |  |  |  |
|  | Shannon | 0.71 |  |  |  |  |  |  |  |  |
|  | Evenness | -0.34 | 0.36 |  |  |  |  |  |  |  |
|  | FRic | 0.63 | 0.58 | -0.20 |  |  |  |  |  |  |
|  | FEve | -0.40 | -0.57 | 0.04 | -0.60 |  |  |  |  |  |
|  | FDiv | -0.74 | -0.24 | 0.38 | -0.23 | -0.19 |  |  |  |  |
|  | RaoQ | -0.42 | 0.29 | 0.91 | 0.04 | -0.18 | 0.55 |  |  |  |
|  | AvTD | -0.16 | -0.50 | -0.56 | -0.55 | 0.30 | -0.20 | -0.73 |  |  |
|  | VarTD | -0.07 | 0.46 | 0.51 | 0.46 | -0.65 | 0.58 | 0.81 | -0.82 |  |
| Chayu | SRic |  |  |  |  |  |  |  |  |  |
|  | Shannon | 0.33 |  |  |  |  |  |  |  |  |
|  | Evenness | 0.01 | 0.94 |  |  |  |  |  |  |  |
|  | FRic | 0.75 | 0.31 | 0.07 |  |  |  |  |  |  |
|  | FEve | 0.33 | 0.65 | 0.56 | 0.30 |  |  |  |  |  |
|  | FDiv | 0.33 | -0.13 | -0.28 | -0.04 | 0.26 |  |  |  |  |
|  | RaoQ | 0.45 | 0.85 | 0.75 | 0.54 | 0.79 | -0.21 |  |  |  |
|  | AvTD | 0.25 | 0.42 | 0.40 | 0.30 | 0.39 | -0.52 | 0.68 |  |  |
|  | VarTD | -0.41 | -0.25 | -0.14 | -0.65 | -0.20 | 0.13 | -0.34 | -0.43 |  |
| Moto | SRic |  |  |  |  |  |  |  |  |  |
|  | Shannon | -0.24 |  |  |  |  |  |  |  |  |
|  | Evenness | -0.70 | 0.83 |  |  |  |  |  |  |  |
|  | FRic | 0.82 | 0.06 | -0.35 |  |  |  |  |  |  |
|  | FEve | -0.45 | -0.17 | 0.15 | -0.46 |  |  |  |  |  |
|  | FDiv | 0.57 | -0.66 | -0.80 | 0.47 | 0.07 |  |  |  |  |
|  | RaoQ | -0.29 | 0.65 | 0.63 | 0.07 | 0.27 | -0.35 |  |  |  |
|  | AvTD | -0.20 | 0.12 | 0.23 | -0.42 | 0.56 | -0.21 | 0.05 |  |  |
|  | VarTD | -0.06 | 0.32 | 0.16 | 0.12 | -0.06 | -0.07 | 0.56 | -0.53 |  |
